# The Outcome of Post-cardiotomy Extracorporeal Membrane Oxygenation in Neonates and Pediatric Patients: A Systematic Review and Meta-Analysis

**DOI:** 10.3389/fped.2022.869283

**Published:** 2022-04-25

**Authors:** Hwa Jin Cho, Insu Choi, Yujin Kwak, Do Wan Kim, Reverien Habimana, In-Seok Jeong

**Affiliations:** ^1^Division of Pediatric Cardiology and Cardiac Critical Care, Department of Pediatrics, Chonnam National University Children's Hospital and Medical School, Gwangju, South Korea; ^2^Cardiovascular and Respiratory Research Team, Chonnam National University Hospital, Gwangju, South Korea; ^3^Department of Thoracic and Cardiovascular Surgery, Chonnam National University Hospital and Medical School, Gwangju, South Korea; ^4^Department of Biomedical Sciences, College of Medicine, Chonnam National University Graduate School, Gwangju, South Korea

**Keywords:** post-cardiotomy, extracorporeal membrane oxygenation, cardiac surgery, neonates, pediatric

## Abstract

**Objective:**

Post-cardiotomy extracorporeal membrane oxygenation (PC-ECMO) is a known rescue therapy for neonates and pediatric patients who failed to wean from cardiopulmonary bypass (CPB) or who deteriorate in intensive care unit (ICU) due to various reasons such as low cardiac output syndrome (LCOS), cardiac arrest and respiratory failure. We conducted a systematic review and meta-analysis to assess the survival in neonates and pediatric patients who require PC-ECMO and sought the difference in survivals by each indication for PC-ECMO.

**Design:**

Systematic review and meta-analysis.

**Setting:**

Multi-institutional analysis.

**Participants:**

Neonates and pediatric patients who requires PC- ECMO.

**Interventions:**

ECMO after open-heart surgery.

**Results:**

Twenty-six studies were included in the analysis with a total of 186,648 patients and the proportion of the population who underwent PC-ECMO was 2.5% (2,683 patients). The overall pooled proportion of survival in this population was 43.3% [95% Confidence interval (CI): 41.3–45.3%; *I*^2^: 1%]. The survival by indications of PC-ECMO were 44.6% (95% CI: 42.6–46.6; *I*^2^: 0%) for CPB weaning failure, 47.3% (95% CI: 39.9–54.7%; *I*^2^: 5%) for LCOS, 37.6% (95% CI: 31.0–44.3%; *I*^2^: 32%) for cardiac arrest and 47.7% (95% CI: 32.5–63.1%; *I*^2^: 0%) for respiratory failure. Survival from PC-ECMO for single ventricle or biventricular physiology, was reported by 12 studies. The risk ratio (RR) was 0.74 for survival in patients with single ventricle physiology (95% CI: 0.63–0.86; *I*^2^: 40%, *P* < 0.001). Eight studies reported on the survival after PC-ECMO for genetic conditions. The RR was 0.93 for survival in patients with genetic condition (95% CI: 0.52–1.65; *I*^2^: 65%, *P* = 0.812).

**Conclusions:**

PC-ECMO is an effective modality to support neonates and pediatric patients in case of failed CPB weaning and deterioration in ICU. Even though ECMO seems to improve survival, mortality and morbidity remain high, especially in neonates and pediatric patients with single ventricle physiology. Most genetic conditions alone should not be considered a contraindication to ECMO support, further studies are needed to determine which genetic abnormalities are associated with favorable outcome.

## Introduction

The use of extracorporeal membrane oxygenation (ECMO) in neonatal and pediatric patients after surgery for congenital heart diseases (CHD) has been steadily increasing as CHD surgery challenges more complex anatomy (1–5).

The role of post-cardiotomy ECMO (PC-ECMO) for neonates and children with cardiorespiratory failure after cardiac surgery for CHD is well-established. PC-ECMO may be required for those who fail to separate from cardiopulmonary bypass (CPB) or who deteriorate in an intensive care unit (ICU) after surgery for various reasons such as thrombosis of systemic-to–pulmonary artery shunts in patients with a single ventricle, intractable arrhythmia, low cardiac output syndrome (LCOS), cardiac arrest and respiratory failure (3, 6).

A recent systematic review revealed that after PC-ECMO, survival-to-hospital discharge ranged from 40 to 60% (7). Survival varied by age and weight, with a higher risk of death seen in neonates. The survival rates also varied widely based on the complexity of underlying CHD. Di Nardo et al. reported PC-ECMO provides perioperative support in 10% of patients with hypoplastic left heart syndrome (HLHS) who undergo the Norwood operation (3).

Despite improving techniques in both CHD surgery and ECMO management, the mortality in neonates and pediatric patients requiring ECMO following CHD surgery remains high, and the survival rate has not changed for the last several decades (5). In postoperative neonates and pediatric patients supported with ECMO, there is a shortage of clinical trials and no meta-analysis to help determine outcome data in select subgroups. As a result, the outcome of benefits of PC-ECMO in neonates and pediatric patients has limited evidence to inform clinical practice and needs to be elucidated. In addition, it is essential to recognize that earlier initiation of ECLS facilitates myocardial recovery and reduces the risk of cardiovascular collapse; however, there is no consensus regarding the optimal timing for ECMO initiation in the perioperative period (3).

Hence, we sought to systematically review the literature to assess survival rates of neonatal and pediatric patients requiring ECMO following CHD surgery and subgroup survival rates by the reason requiring ECMO in published cohorts.

## Methods

### Search Strategy

A systematic search was performed in Medline, Embase and Cochrane Library. We searched for English-written studies from January 2010 to December 2021. This systematic review and meta-analysis was registered in the International prospective register of systematic reviews PROSPERO under the registration number: CRD42022295819. We adopted the search strategy following keywords and their variations; (1) population 1: “Child”, OR “Pediatrics” OR “Infant” OR “Neonate” and others, (2) population 2: “Cardiac Surgical Procedures” OR “Thoracic Surgery” OR “Cardiopulmonary Bypass” and others, (3) Intervention: “Extracorporeal Membrane Oxygenation” and others (Supplementary File 1).

### Inclusion and Exclusion Criteria

**Figure 1:**
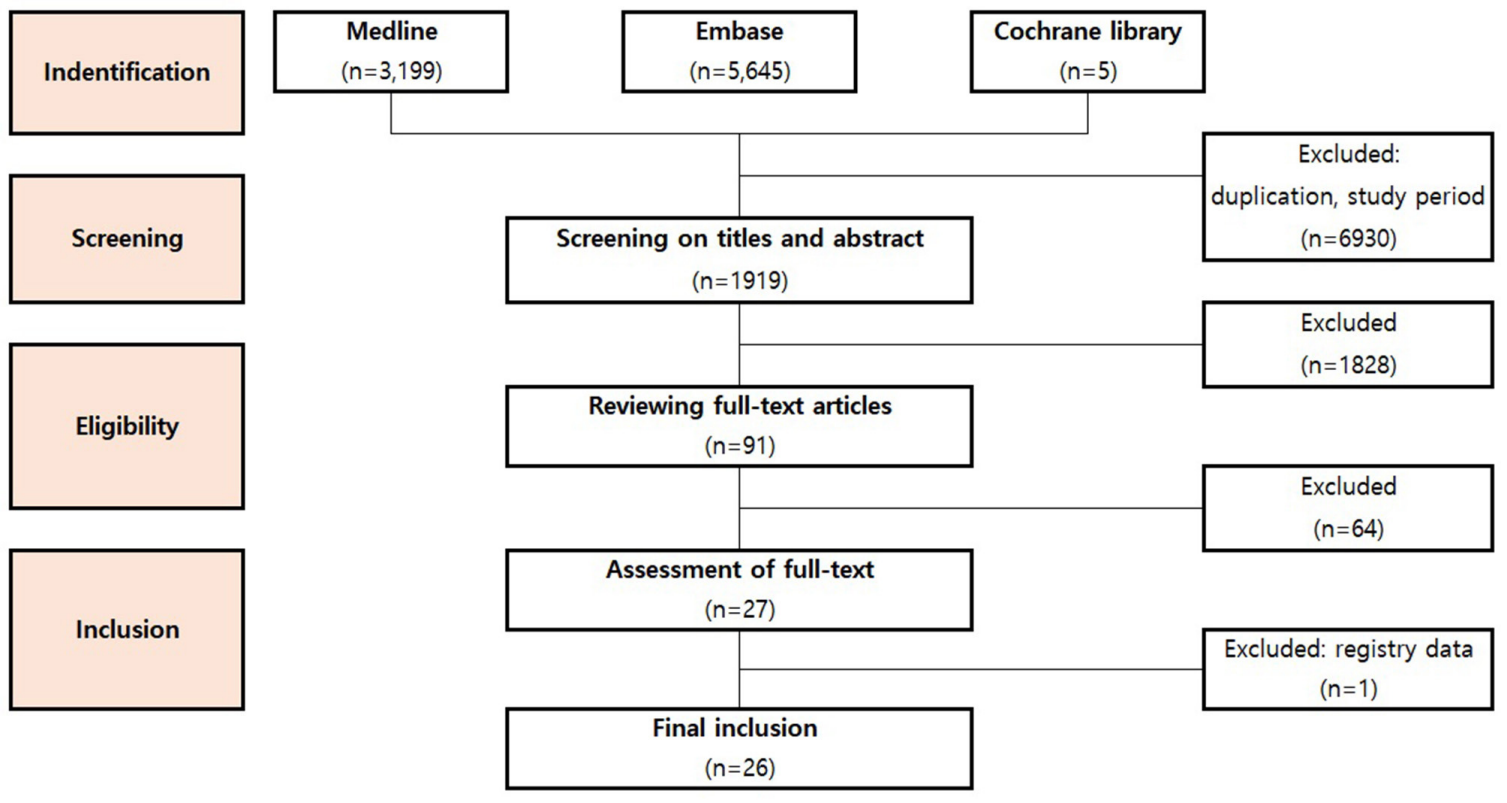
Preferred reporting items for systematic reviews and meta-analyses (PRISMA) flow chart.

We searched and enrolled English articles that present the outcome of PC-ECMO in the neonate and/or pediatric patients, but only those which met the Population, Interventions, Comparison and Outcomes (PICO) criteria (Supplementary File 2) were included in the analysis. The authors followed the guidelines for Preferred Reporting Items for Systematic reviews and Meta-Analyses (PRISMA) (8). All three independent reviewers reviewed all articles (HJC, RH, ISJ) to screen titles and abstracts based on predefined inclusion criteria. The full text of the eligible articles was reviewed. Three reviewers extracted and compared information of all included articles and evaluated all the studies. When they disagreed, disagreements were either discussed to reach a consensus between the two reviewers or decided by a third reviewer. Selected studies also had to fulfill the following inclusion criteria: (1) provide data on neonates and/or pediatric patients who required PC-ECMO; (2) include patients aged 18 years or younger; (3) report data on in-hospital mortality; (4) be a prospective or retrospective observational study; (5) published in the English language as a full article; (6) include at least ten patients; (7) published since 2011. Studies were excluded if they (1) did not report information on the use of PC-ECMO or (2) did not provide data on the outcome (3) included only adult patients. Studies published in languages other than English (4) and those published before 2010 (5), were omitted. Review articles, animal studies, conferences, abstracts, meetings, studies with <5 in sample size, studies with unclear information and data from registry were also excluded (6) (Figure 1).

### Data Extraction and Outcome Measures

The retrieved articles were independently collected and checked by three researchers (HJC, RH, ISJ). The involvement of a third investigator settled any disagreement on collected data to help reach the consensus. There was no attempt to obtain specific or missing data from the authors. The following data were extracted: first author, year of publication, the overall number of cardiac surgery procedures performed during the study period, type of intervention, number of patients supported with PC-ECMO, gender, significant outcomes. The quality of the included studies was assessed by using RoBANS (Risk of Bias Assessment tool for Non-randomized Studies) (9). The RoBANS contains six domains, including (1) the selection of participants, (2) confounding variables, (3) measurement of intervention (exposure), (4) blinding of outcome assessment, (5) incomplete outcome data, and (6) selective outcome reporting. The primary outcome of this analysis was pooled proportional survival to hospital discharge in this cohort. The secondary outcomes were pooled survival of subgroup by each indication for PC-ECMO, pooled survival of neonatal ECMO, pooled survival differences between the single ventricle and biventricular physiology, pooled survival difference between genetic and non-genetic conditions (Supplementary File 3).

### Statistical Analysis

We analyzed the data using R (Version 3.6.3, R Foundation for Statistical Computing, Vienna, Austria) and Rex software (Version 3.6.3, RexSoft Inc., Seoul, South Korea). We showed different pooled proportional rate or risk ratio (RR) with 95% confidence interval (CI), using a forest plot with a fixed and random-effects model. Additionally, the possibility of publication bias was checked through a funnel plot. Cochran Q tests and *I*^2^ were used to evaluate the possible heterogeneity. *I*^2^ < 25% was considered as of low heterogeneity, 25–70% was of mediate heterogeneity and >70% was of high heterogeneity. If *p*-value > 0.1 and *I*^2^ < 50%, the fixed effects model was selected; otherwise, the heterogeneity was assessed to determine whether the random effects model could be used. *p*-value < 0.05 was considered statistically significant.

## Results

Twenty-six studies (2, 10–34) were included in the analysis with a total of 186,648 patients, and the proportion of the population who underwent PC-ECMO was 2.5% (26 studies reporting on 2,683 patients) (Table 1).

**Table 1:** Demographics of the included studies.

	**References**	**Year**	**Country**	**Sample size**	**Survivor**	Age^*^	**Main issues**
1	Ugaki et al. (33)	2010	Japan	12	5	0.9	Norwood stage 1 procedures
2	Polimenakos et al. (30)	2011	USA	14	6	0.26	Single ventricle physiology
3	Itoh et al. (22)	2012	Japan	76	28	10.8	Developmental outcomes
4	Wolf et al. (34)	2012	USA	90	50		ECPR
5	Bhat et al. (14)	2013	USA	64	21	0.22	Body weight <3 kg
6	Alsoufi et al. (11)	2014	Saudi Arabia	39	16	2.4	Rapid response ECPR
7	Alsoufi et al. (12)	2014	Saudi Arabia	100	37	2.4	Single ventricle physiology
8	Jolley et al. (24)	2014	USA	103	42	5.5	Cavopulmonary shunt
9	Philip et al. (28)	2014	USA	59	27	35	Underlying heart disease
10	Florez et al. (17)	2015	USA	37	12	0.2	Setting Up an ECMO Program
11	Gupta et al. (20)	2015	Colombia	52	27	3.47	ECMO run > 7 days
12	Howard et al. (21)	2016	USA	84	42	0.18	Residual lesion
13	Misfeldt et al. (27)	2016	USA	751	322		Single ventricle physiology
14	Sznycer-Taub et al. (32)	2016	USA	93	35	0.35	Hyperoxia
15	Furlong-Dillard et al. (18)	2017	USA	327	153		Genetic conditions, multicenter
16	Polimenakos et al. (29)	2017	USA	21	10	0.25	After hospital discharge
17	Kuraim et al. (25)	2018	Canada	20	9	0.42	Risk of mortality
18	Achuff et al. (10)	2019	USA	187	86		Risk of mortality
19	Azizov et al. (13)	2019	Germany	45	15	21.71	Improving survival
20	Dohain et al. (15)	2019	Egypt	30	12	9.12	Risk of mortality
21	ElMahrouk et al. (16)	2019	Saudi Arabia	88	42	61.04	Multicenter
22	Guo et al. (19)	2019	China	11	4	6.86	ECPR
23	Merkle et al. (26)	2019	Germany	39	17		Mid- and Long-term survival
24	Ergün et al. (2)	2020	Turkey	133	51	53.35	Improving survival
25	Jin et al. (23)	2021	Australia	85	40	20.76	Early mortality
26	Yu et al. (34)	2021	China	23	12	0.3	Neonatal ECMO

* *Age: mean age (months)*.

*ECMO, extracorporeal membrane oxygenation; ECPR, extracorporeal cardiopulmonary resuscitation; USA, United State of America*.

**Figure 2:**
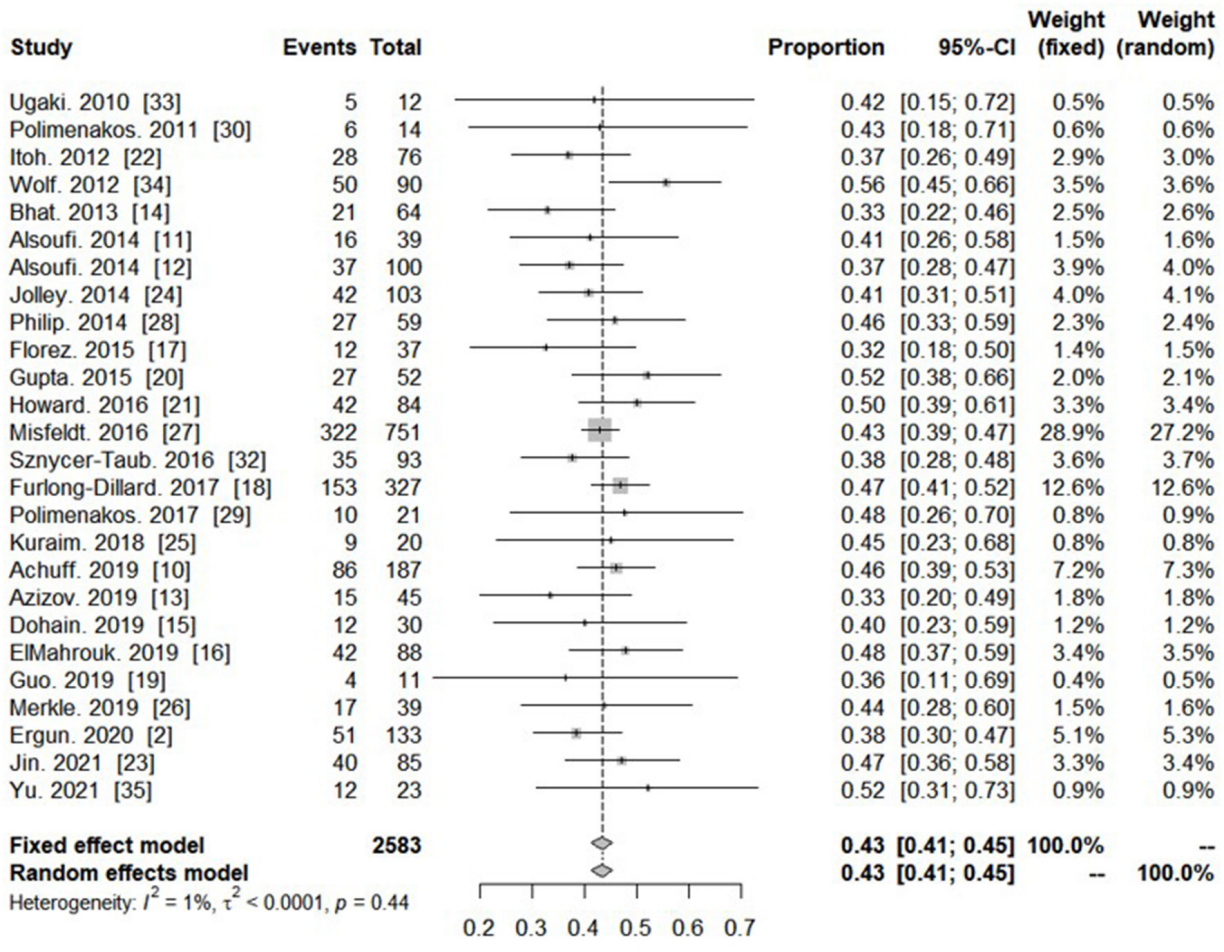
Forest plot showing proportion of survivors with post-cardiotomy extracorporeal membrane oxygenation in all included studies.

**Figure 3:**
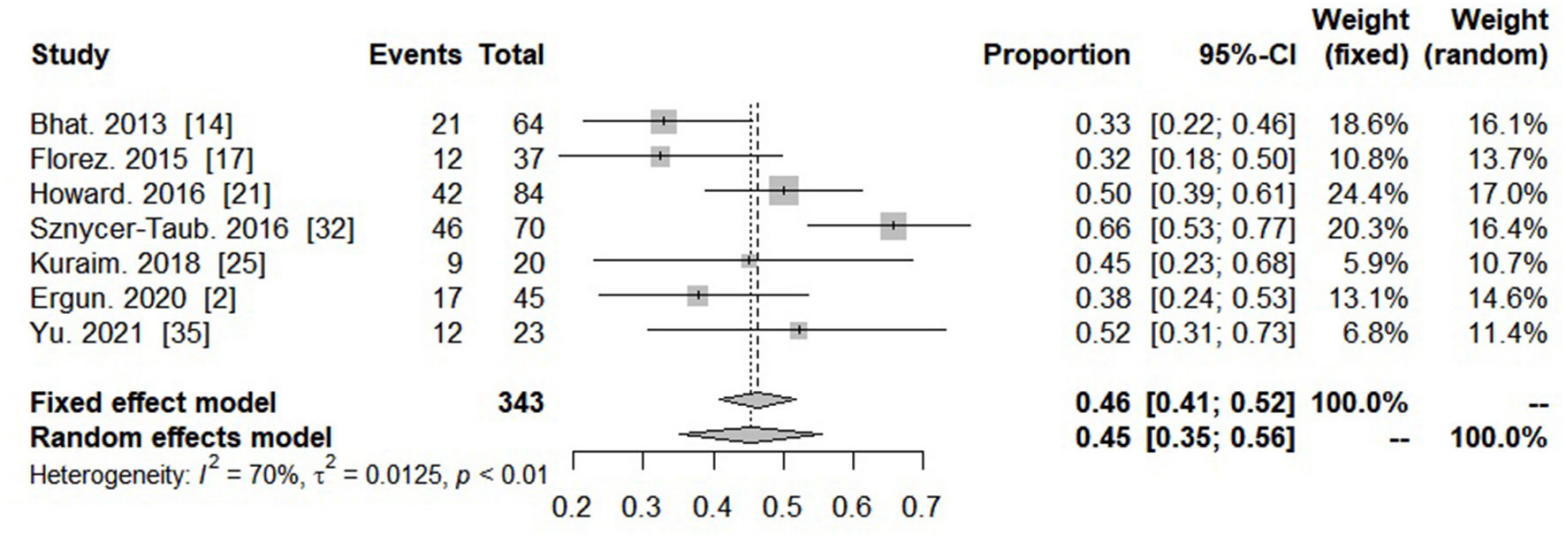
Forest plot showing proportion of survivors in neonates with post-cardiotomy extracorporeal membrane oxygenation.

All twenty-six studies reported on the outcome of patients who required PC-ECMO. The overall pooled proportion of survival in this population was 43.3% (95% CI: 41.3–45.2%, *I*^2^ = 1%) (Figure 2; Supplementary File 4). Among total included studies, seven studies reported on the survival rate of PC-ECMO in neonates. The pooled survival rate was 43.7% (95% CI: 35.4–52.2%; *I*^2^: 77%) (Figure 3).

### Survival Outcome by Indications

**Figure 4:**
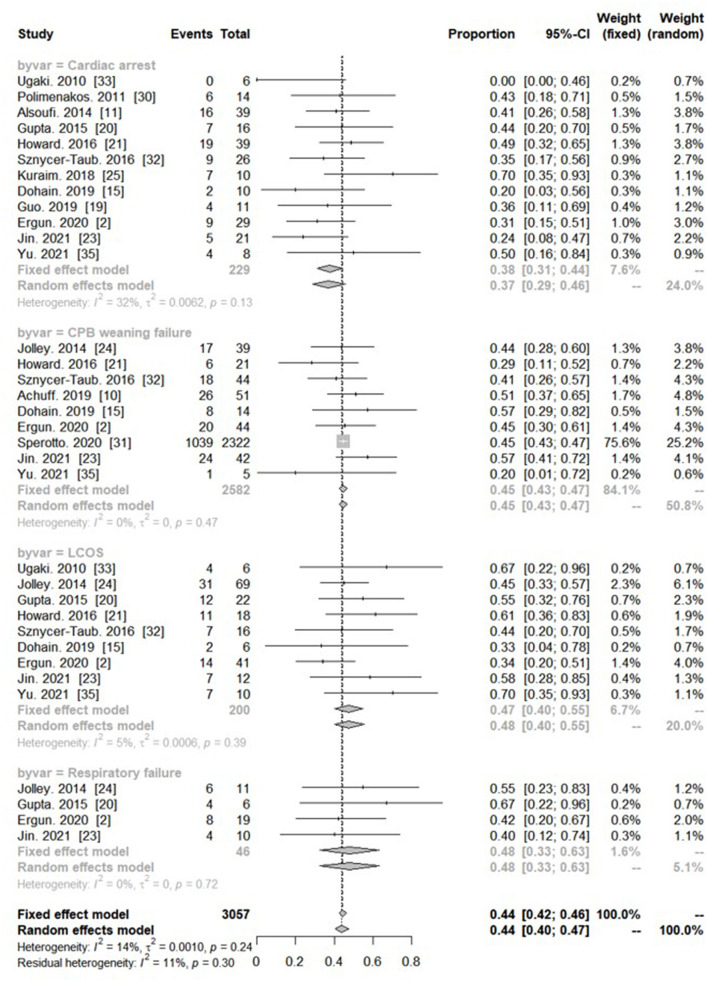
Forest plot showing subgroup analysis of proportional survival in different indications with post-cardiotomy extracorporeal membrane oxygenation.

Nine studies reported on the outcome of CPB weaning failure. The pooled survival rate of patients with CPB weaning failure was 44.7% (95% CI: 42.6–46.7; *I*^2^:0%). Nine studies reported on the outcome of LCOS. The pooled survival rate was 47.3% (95% CI: 39.9–54.7%; *I*^2^: 5%). Twelves studies reported on the outcome of PC-ECMO for cardiac arrest. The pooled survival rate was 37.6% (95% CI: 31.0–44.3%; *I*^2^: 32%). Four studies reported survival rate after PC-ECMO for respiratory failure. The pooled survival rate was 47.7% (95% CI: 32.5–63.1%; *I*^2^: 0%). But there was no significant difference in pooled survival in subgroup analysis, based on each indication of PC-ECMO support (interaction between groups. *P* = 0.19) (Figure 4).

### Relative Risk of Survival With Single Ventricle Physiology and Genetic Condition

**Figure 5:**
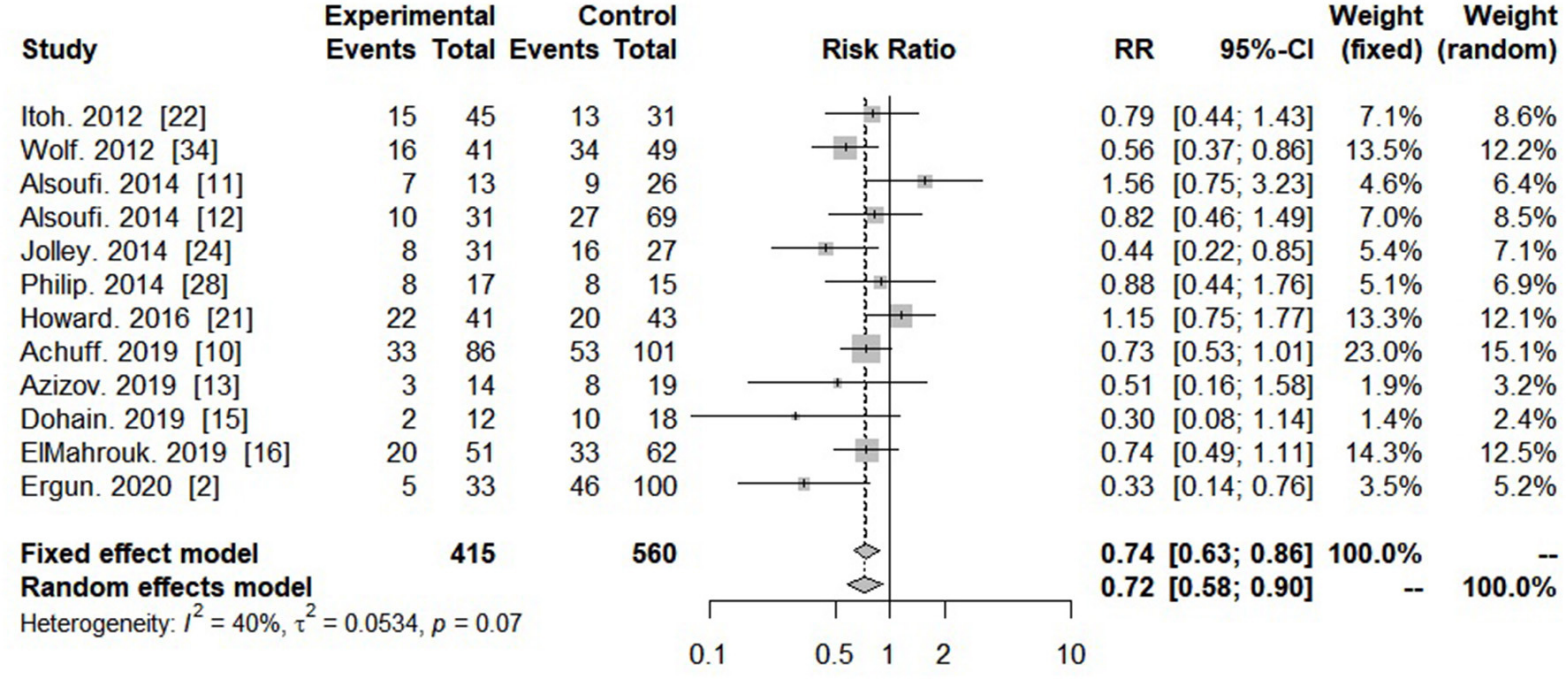
Forest plot showing risk ratio of survival in single ventricle or biventricular physiology with post-cardiotomy extracorporeal membrane oxygenation.

Survival from PC-ECMO for single ventricle or biventricular physiology was reported by 12 studies. Patients with single ventricle physiology had a significantly lower survival probability, compared to patients with biventricular physiology (RR = 0.74; 95% CI: 0.63–0.86; *I*^2^ = 40%, *P* < 0.001) (Figure 5).

**Figure 6:**
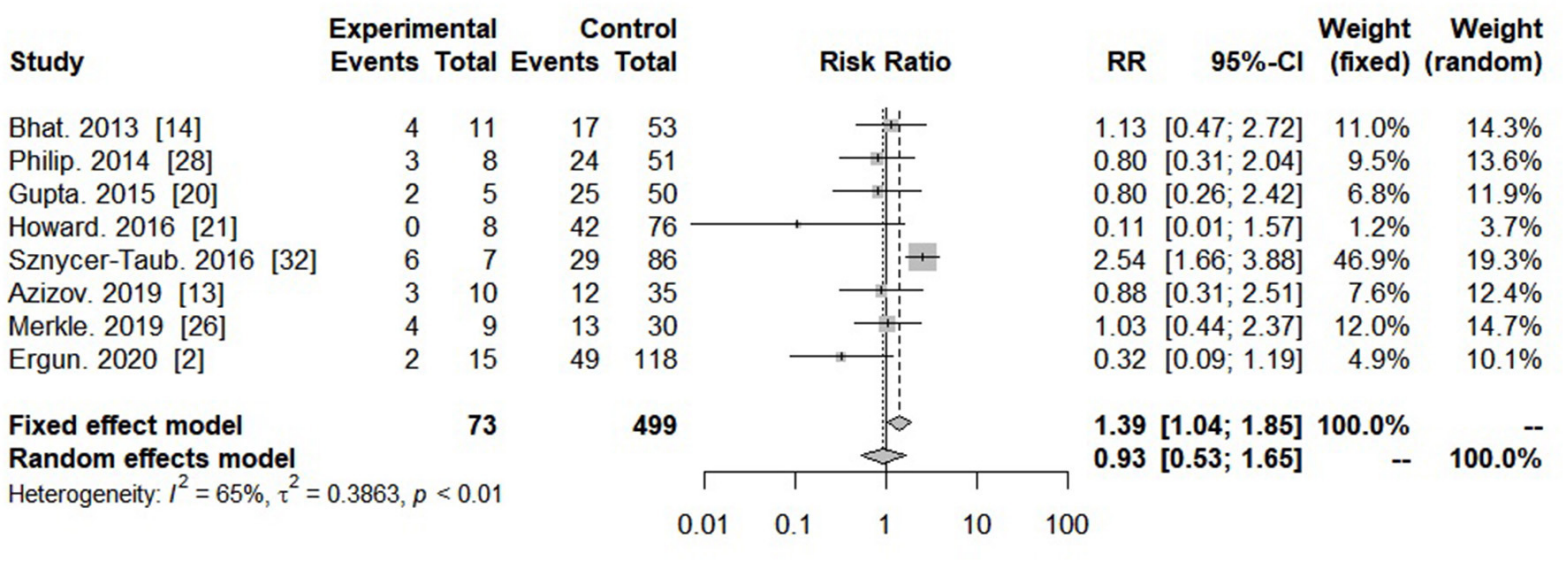
Forest plot showing risk ratio of survival in genetic or non-genetic condition with post-cardiotomy extracorporeal membrane oxygenation.

Eight studies reported the survival rate of PC-ECMO in patients with genetic conditions. Patients with genetic condition had a similar survival probability, compared to patients without genetic condition (RR = 0.93, 95% CI: 0.53–1.65, *I*^2^: 65%, *P* = 0.812) (Figure 6).

## Discussion

PC-ECMO has long been established to support neonate and pediatric patients who fail to separate from CPB and/or those who deteriorate in ICU with variable survival rates (7). Our overall survival rate of PC-ECMO of 43%. The survival in neonates and pediatric patients requiring ECMO following CHD surgery remains high; it is comparable to the lower end of survival to discharge of ELSO registry (2011–2020) data which is 43–63% for cardiac failure.

### Survival Outcome by Indications

Our meta-analyses indicate that the survival rate varies according to the conditions before PC-ECMO application. While 46–47% of patients who failed to wean from CPB and deteriorated in ICU due to low cardiac output syndrome and respiratory failure survived, those, who required ECMO for cardiac arrest showed the lowest survival rate (39%). In neonatal and pediatric patients with LCOS refractory to medical management, earlier initiation of ECLS facilitates unloading of myocardial burden and reduces the risk of cardiovascular collapse (3). While there is no consensus on the timing for ECMO initiation in the perioperative period, our meta-analyses showed that neonates and pediatric patients with LCOS supported with ECMO had lower mortality than patients with ECMO after cardiac arrest (ECPR).

Respiratory failure overall has a survival rate of 59–72%, according to the ELSO registry. However, the survival of PC-ECMO for respiratory failure in our meta-analysis was observed lower survival. Despite advancement in surgical technique, 2–30% of pediatric patients required ECMO support for hypoxia or respiratory distress (5, 7, 35). Although there is limited data regarding PC-ECMO for respiratory failure, four studies with 46 patients were included in our meta-analyses with a survival rate of 48%. Likely neonates and pediatric patients recovering from surgery for CHD have higher disease severity than the entire cohort of respiratory failure patients supported with respiratory ECMO.

### Single Ventricle Physiology

The complexity of the underlying CHD and cardiac surgery for CHD is an essential determinant of survival to hospital discharge. As the complexity varies, the survival hospital discharge of those patients requiring ECMO also varies widely (20, 30, 36). Our meta-analysis included 560 patients with biventricular physiology reported by 12 studies. Single ventricle physiology was associated with a lower pooled survival rate than biventricular physiology (35.9 vs. 49.5%). Mascio et al. demonstrated analysis of children with perioperative mechanical circulatory support (MCS) by the Society of Thoracic Surgeons Congenital Heart Surgery Database. The operations with the highest MCS rate were the Norwood procedure (17%), and 53.2% of the MCS patients did not survive hospital discharge after cardiac surgery (37). Misfeldt et al. have reported admission rate of single ventricle patients requiring ECMO was 2.3% of all hospitalization, and the mortality rate of this population was 57.1%, with no change over time (27).

### Genetic Conditions

Recognizable syndromes, extracardiac malformations, and chromosomal anomalies associated with genetic conditions occur in ~20–30% of children with CHD (38). Over time, the exclusion strategy of genetic abnormalities from cardiac surgical palliation has changed (39); previously, physicians were more reluctant to provide ECMO to this population, but the view has been changed over time that with changing outcome and even ECMO in patients with Trisomy 13 or 18 is has been reported in the recent era (39, 40). In our meta-analysis, eight studies with data of genetic conditions were included. The neonates and pediatric patients with genetic conditions tend to be a similar survival outcome with a RR of 0.93 compared to those without genetic condition. Although limited literature was found regarding genetic abnormalities and CHD requiring ECMO, Furlong-Dillard JM et al. have reported 327 patients with genetic abnormalities who required ECMO after CHD surgery with the mortality rate of 2–3% (18). Alsoufi et al. reported that despite adjustment for prematurity and low birth weight, children with genetic abnormalities had a significantly increased risk of death after cardiac surgery compared to children without genetic conditions (41–44).

### Study Limitations

There are several limitations in our meta-analysis. First, this analysis included articles published during the last 10 years in an attempt to mitigate advancements in technology. However, there is a possibility that we could have missed some valuable and historical data from earlier studies. Second, we did not include studies with cohorts of a bridge to transplantation and preoperative ECMO for CHD. Third, we didn't collect the specific indications and conditions, such as timing/severity of illness, individual cardiac anomalies, and detailed genetic information. Lastly, all included articles are retrospective observational studies, while randomized control studies could have yielded better quality of our findings.

## Conclusions

PC-ECMO is an effective modality to support neonates and pediatric patients in case of failed CPB weaning and deterioration in ICU. Even though ECMO seems to improve survival, mortality and morbidity remain high, especially in neonates and pediatric patients with single ventricle physiology. Most genetic conditions alone should not be considered a contraindication to ECMO support, further studies are needed to determine which genetic abnormalities are associated with favorable outcome.

## Data Availability Statement

**T**he raw data supporting the conclusions of this article will be made available by the authors, without undue reservation.

## Author Contributions

HC and I-SJ: concept and design, data analysis, and data interpretation. HC, RH, and I-SJ: PROSPERO registration and data collection. HC: manuscript write up and manuscript formatting. RH and I-SJ: tables and figures. HC, IC, YK, DK, RH, and I-SJ: manuscript editing. All authors read and approved the final manuscript.

## Funding

This work was supported by grants BCRI19255 and BCRI21043 from the Chonnam National University Hospital Biomedical Research Institute and by grants NRF-2019R1D1A3A03103899 and NRF-2020R1F1A1073921 from the Ministry of Education of the Republic of Korea and National Research Foundation of Korea to HC and I-SJ.

## Conflict of Interest

The authors declare that the research was conducted in the absence of any commercial or financial relationships that could be construed as a potential conflict of interest.

## Publisher's Note

All claims expressed in this article are solely those of the authors and do not necessarily represent those of their affiliated organizations, or those of the publisher, the editors and the reviewers. Any product that may be evaluated in this article, or claim that may be made by its manufacturer, is not guaranteed or endorsed by the publisher.
