# Supplementary material for: The Outcome of Post-cardiotomy Extracorporeal Membrane Oxygenation in Neonates and Pediatric Patients: A Systematic Review and Meta-Analysis
Source: Front Pediatr. 2022 Apr 25;10:869283. doi: 10.3389/fped.2022.869283 (PMC9083359; doi:10.3389/fped.2022.869283)
Supplement: Supplementary file 1 [file Data_Sheet_1.docx]

**Supplementary file 1.** Search strategy in each database.

| **Pubmed** | | |
| --- | --- | --- |
| #1 | Extracorporeal Membrane Oxygenation [Mesh] | 13,397 |
| #2 | Extracorporeal Membrane Oxygenation* [TIAB] OR Extracorporeal Life Support [TIAB] OR ECMO [TIAB] OR ECLS [TIAB] OR Extracorporeal Circulation* [TIAB] OR Extracorporeal [TIAB] | 47,175 |
| #3 | #1 OR #2 | 535,278 |
| #4 | Cardiac Surgical Procedures [Mesh] OR Thoracic Surgery [Mesh] OR Cardiopulmonary Bypass [Mesh] OR Heart Defects, Congenital [Mesh] OR Fontan Procedure [Mesh] OR Norwood Procedures [Mesh] OR Hypoplastic Left Heart Syndrome [Mesh] OR Univentricular Heart [Mesh] OR Transposition of Great Vessels [Mesh] | 514,233 |
| #5 | Cardiac Surgical Procedure* [TIAB] OR Thoracic Surgery [TIAB] OR Cardiopulmonary Bypass [TIAB] OR Heart Defect* [TIAB] OR Fontan [TIAB] OR Norwood [TIAB] OR Hypoplastic Left Heart Syndrome [TIAB] OR Univentri* [TIAB] OR Single ventri* [TIAB] OR Transposition of Great Arter* [TIAB] | 78,484 |
| #6 | #4 OR #5 | 48,977 |
| #7 | Child [Mesh] OR Pediatrics [Mesh] OR Infant [Mesh] OR Infant, Newborn [Mesh] OR Adolescent [Mesh] | 3,834,544 |
| #8 | Child* [TIAB] OR Pediatric* [TIAB] OR Infant* [TIAB] OR Newborn*[TIAB] OR Adolescent* [TIAB] | 2,259,270 |
| #9 | #7 OR #8 | 4,401,215 |
| #10 | #3 AND #6 AND #9 | 3,199 |

| **Embase** | | |
| --- | --- | --- |
| #1 | child'/exp OR 'child' OR 'children' OR 'newborn'/exp OR 'animals, newborn' OR 'child, newborn' OR 'full term infant' OR 'human neonate' OR 'human newborn' OR 'infant, newborn' OR 'neonatal animal' OR 'neonate' OR 'neonate animal' OR 'neonatus' OR 'newborn' OR 'newborn animal' OR 'newborn animals' OR 'newborn baby' OR 'newborn child' OR 'newborn infant' OR 'newly born animal' OR 'newly born baby' OR 'newly born child' OR 'newly born infant' OR 'infant'/exp OR 'infant' OR 'pediatric'/exp OR 'adolescent'/exp OR 'adolescent' OR 'teenager' | 4,886,045 |
| #2 | extracorporeal oxygenation'/exp OR 'ecls (extracorporeal life support)' OR 'ecls therapy' OR 'ecls treatment' OR 'ecmo (extracorporeal membrane oxygenation)' OR 'ecmo support' OR 'ecmo therapy' OR 'ecmo treatment' OR 'extra corporal membrane oxygenation' OR 'extra corporeal life support' OR 'extra corporeal membrane oxygen support' OR 'extra corporeal membrane oxygenation' OR 'extra corporeal membrane oxygenator support' OR 'extra corporeal membrane oxygenator therapy' OR 'extra corporeal membranous oxygenation' OR 'extra corporeal oxygenation' OR 'extra-corporeal membrane oxygena-tion' OR 'extra-pulmonary oxygen therapy' OR 'extra-pulmonary oxygenation' OR 'extra-pulmonic oxygenation' OR 'extracorporal membrane oxygenation' OR 'extracorporal oxygenation' OR 'extracorporal oxygenization' OR 'extracorporeal circulation membrane oxygen support' OR 'extracorporeal life support' OR 'extracorporeal membran-oxygenation' OR 'extracorporeal membrane oxygen (therapy)' OR 'extracorporeal membrane oxygen support' OR 'extracorporeal membrane oxygen-ation' OR 'extracorporeal membrane oxygenation' OR 'extracorporeal membrane oxygenaton' OR 'extracorporeal membraneous oxygenation' OR 'extracorporeal membranes oxygenation' OR 'extracorporeal membranooxygenation' OR 'extracorporeal membranous oxygen support' OR 'extracorporeal membranous oxygenation' OR 'extracorporeal membranous oxygenator support' OR 'extracorporeal membranoxygenation' OR 'extracorporeal oxygenation' OR 'extracorporeal pump oxygenation' OR 'extrapulmonary blood oxygenation' OR 'extrapulmonary membrane oxygenation' OR 'extrapulmonary oxygen therapy' OR 'extrapulmonary oxygenation' OR 'membrane oxygenation, extracorporeal' OR 'oxygenation, extracorporeal' | 38,909 |
| #3 | postcardiotomy OR 'heart surgery'/exp OR 'cardiac surgery' OR 'cardiac surgical procedures' OR 'cardiosurgery' OR 'heart operation' OR 'heart surgery' OR 'myocardial resection' OR 'surgery, heart' OR 'thorax surgery'/exp OR 'cardiothoracic surgery' OR 'chest surgery' OR 'chest wall surgery' OR 'surgery, chest' OR 'surgery, thoracic' OR 'surgery, thorax' OR 'thoracic operation' OR 'thoracic surgery' OR 'thoracic surgical procedures' OR 'thorax surgery' OR 'cardiopulmonary bypass'/exp OR 'atriopulmonary shunt' OR 'bypass, cardiopulmonary' OR 'cardiopulmonary bypass' OR 'cardiopulmonary shunt' OR 'heart lung bypass' OR 'heart defect'/exp OR 'fontan procedure'/exp OR 'fontan anastomosis' OR 'fontan conduit' OR 'fontan connection' OR 'fontan connections' OR 'fontan operation' OR 'fontan operations' OR 'fontan procedure' OR 'fontan procedures' OR 'norwood procedure'/exp OR 'norwood operation' OR 'norwood palliation' OR 'norwood palliative procedure' OR 'norwood palliative surgery' OR 'norwood procedure' OR 'norwood procedures' OR 'norwood surgery' OR 'norwood technique' OR 'cavopulmonary connection'/exp OR 'cavo-pulmonary anastomoses' OR 'cavo-pulmonary anastomosis' OR 'cavo-pulmonary connection' OR 'cavo-pulmonary shunt' OR 'cavo-pulmonary shunts' OR 'cavopulmonary anastomoses' OR 'cavopulmonary anastomosis' OR 'cavopulmonary connection' OR 'cavopulmonary shunt' OR 'cavopulmonary shunts' OR 'hypoplastic left heart syndrome'/exp OR 'congenital heart disease'/exp OR 'congenital cardiac disease' OR 'congenital cardiac distress' OR 'congenital heart disease' OR 'congenital heart distress' OR 'congenital heart failure' OR 'heart congenital disease' OR 'heart disease, congenital' OR 'neonatal cardiopathy' OR 'truncus arteriosus, persistent' OR 'great vessels transposition'/exp OR 'great artery transposition' OR 'great vessel transposition' OR 'great vessels transposition' OR 'large vessel transposition' OR 'transposition of great vessels' OR 'transposition, great vessels' | 1,016,916 |
| #4 | #1 AND #2 AND #3 | 5,645 |

| **Cochrane Library** | | |
| --- | --- | --- |
| #1 | MeSH descriptor: [Extracorporeal Membrane Oxygenation] explode all trees OR (ECMO):ti,ab,kw | 100 |
| #2 | MeSH descriptor: [Child] explode all trees OR MeSH descriptor: [Infant] explode all trees OR MeSH descriptor: [Infant, Newborn] explode all trees OR (neonate):ti,ab,kw OR (infant):ti,ab,kw OR ("Child"):ti,ab,kw | 202,826 |
| #3 | MeSH descriptor: [Thoracic Surgery] explode all trees OR MeSH descriptor: [Cardiopulmonary Bypass] explode all trees OR MeSH descriptor: [Heart Defects, Congenital] explode all trees OR MeSH descriptor: [Fontan Procedure] explode all trees OR MeSH descriptor: [Heart Bypass, Right] explode all trees OR (thoracic surgery):ti,ab,kw OR (cardaic surgery):ti,ab,kw OR (congenital heart disease):ti,ab,kw OR ("Norwood"):ti,ab,kw OR ("Fontan"):ti,ab,kw | 13,333 |
| #4 | #1 AND #2 AND #3 | 5 |
