# Supplementary material for: The Outcome of Post-cardiotomy Extracorporeal Membrane Oxygenation in Neonates and Pediatric Patients: A Systematic Review and Meta-Analysis
Source: Front Pediatr. 2022 Apr 25;10:869283. doi: 10.3389/fped.2022.869283 (PMC9083359; doi:10.3389/fped.2022.869283)
Supplement: Supplementary file 2 [file Data_Sheet_2.docx]

**Supplementary file 2.** Population, intervention, comparison, and outcomes (PICO) description for meta-analysis

| **Population** | Neonate and/or Children who underwent cardiac surgery |
| --- | --- |
| **Intervention** | Post-cardiotomy extracorporeal membrane oxygenation |
| **Comparison** | None |
| **Outcomes** | Survivals by various indications or conditions |
