# Supplementary material for: The Outcome of Post-cardiotomy Extracorporeal Membrane Oxygenation in Neonates and Pediatric Patients: A Systematic Review and Meta-Analysis
Source: Front Pediatr. 2022 Apr 25;10:869283. doi: 10.3389/fped.2022.869283 (PMC9083359; doi:10.3389/fped.2022.869283)

Supplementary file 3. Assessment of risk of bias with RoBANS. A: Summary, B: Detailed figure with a low risk of bias (+), an unclear risk of bias (blank) and a high risk of bias (-).

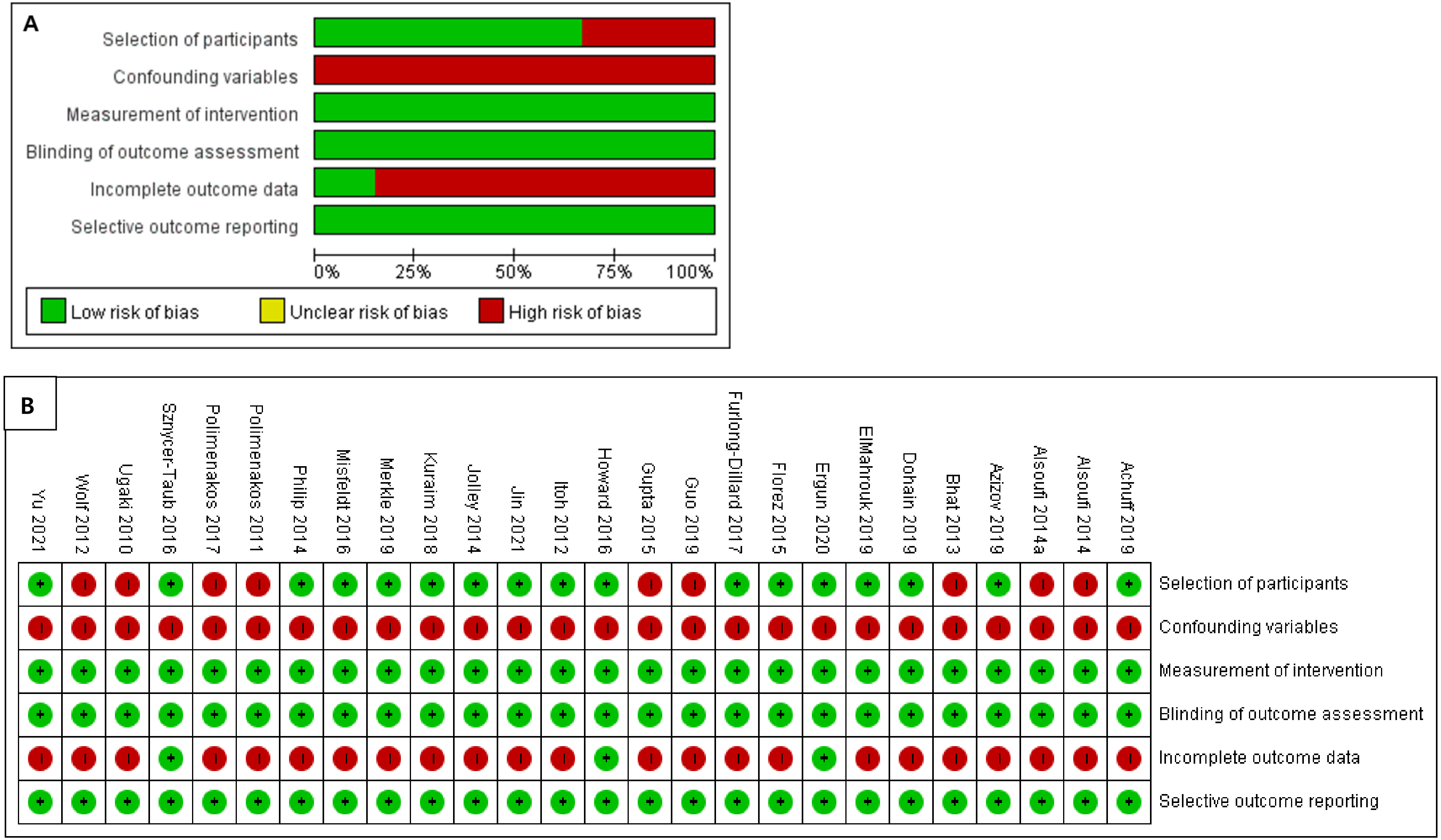

Supplement: Supplementary file 3 [file Data_Sheet_3.pdf]
