# Supplementary figures and images for: The Outcome of Post-cardiotomy Extracorporeal Membrane Oxygenation in Neonates and Pediatric Patients: A Systematic Review and Meta-Analysis
Source: Front Pediatr. 2022 Apr 25;10:869283. doi: 10.3389/fped.2022.869283 (PMC9083359; doi:10.3389/fped.2022.869283)

Supplementary file 4. Funnel plot for all included studies.

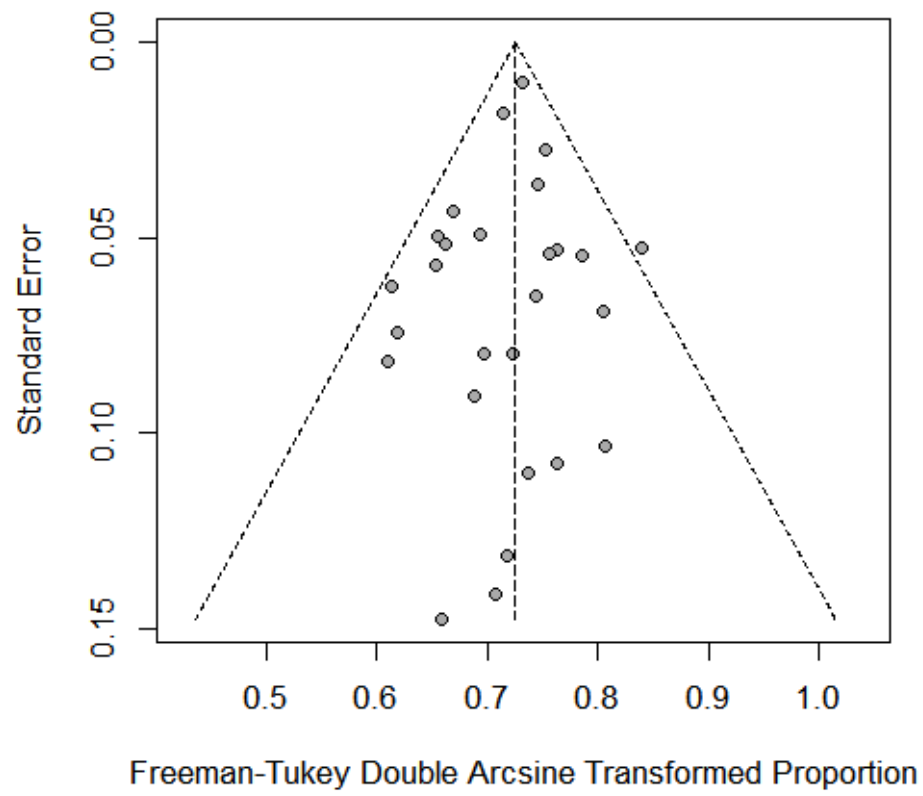

Supplement: Supplementary file 4 [file Data_Sheet_4.pdf]
